# Higher pedicle screw density does not improve curve correction in Lenke 2 adolescent idiopathic scoliosis

**DOI:** 10.1186/s13018-021-02415-4

**Published:** 2021-04-21

**Authors:** Timothy J. Skalak, Joel Gagnier, Michelle S. Caird, Frances A. Farley, Ying Li

**Affiliations:** 1 Nationwide Children’s Hospital, Columbus, OH USA; 2 Columbus, USA; 3 C.S. Mott Children’s Hospital, University of Michigan Health System, Ann Arbor, MI USA

**Keywords:** Scoliosis, Implant density, Spinal deformity, Lenke 2

## Abstract

**Purpose:**

Higher pedicle screw density posterior spinal fusion (PSF) constructs have not been shown to result in improved curve correction in Lenke 1 and 5 adolescent idiopathic scoliosis (AIS) but do increase cost. The purpose of this study questioned whether higher screw density constructs improved curve correction and maintenance of correction in Lenke 2 AIS. Secondary goals were to identify predictive factors for correction and postoperative magnitude of curves in Lenke 2 AIS.

**Methods:**

We identified patients 11 to 17 years old who underwent primary PSF for Lenke 2 AIS between 2007 and 2017 who had minimum follow-up of 2 years. Demographic and radiographic data were collected to perform regression and elimination analysis.

**Results:**

Thirty patients (21 females, 9 males) were analyzed. Average age and SD at time of surgery was 14.0 ± 1.8 years (range, 11–17 years), and median follow-up was 2.8 years (IQR 2.1–4.0 years). Implant density did not predict final postoperative curve magnitude. Predictors of final postoperative curve magnitude were sex and preoperative curve magnitude. Predictors of percentage of correction of major curve were sex and age at the time of surgery. Predictors of final postoperative thoracic kyphosis were sex and percent flexibility preop. Females had lower final postoperative major curve magnitude, a higher percent curve correction, and lower postoperative thoracic kyphosis.

**Conclusions:**

Increased implant density is not predictive of postoperative curve magnitude in Lenke 2 AIS. Predictors of postoperative curve magnitude are sex and preoperative curve magnitude.

**Level of evidence:**

Level III, retrospective observational

## Introduction

Pedicle screws have become the standard instrumentation for the surgical treatment of adolescent idiopathic scoliosis (AIS) [1–3]. Pedicle screw constructs have demonstrated lower revision surgery rates than hybrid or hook-rod constructs and have been shown to be able to be safely placed in the pediatric population [4, 5]. Theoretically, these stronger implants may allow surgeons to achieve and maintain better overall radiographic curve correction as well as improved clinical outcomes [6]. However, there are potential drawbacks to the use of pedicle screws. Pedicle screw instrumentation has resulted in higher costs for scoliosis surgery [7, 8]. Some previous work has demonstrated cost savings that would occur if fewer pedicle screws could be used safely [8]. In a group of 19 Shriners Hospitals, the implant cost varied from $4092 for an all hook construct to up to $22,824 for an all pedicle screw construct. In addition, the cost of identical variable angle pedicle screws varied between hospitals from $753 to $1215 per screw. The reasons for these variations are likely multifactorial, but they highlight the potential financial implications of implant use. In an era of increasingly cost-conscious care, these increased costs must be justified by radiographic and, more importantly, clinical benefits. Further, multiple studies have demonstrated significant rates of screw malposition even in experienced hands with the rate of malpositioned screws in spinal deformity surgery estimated in several studies to be between 5.1 and 9% [9–11], with higher incidences of screw malposition associated with upper thoracic level instrumentation. Malpositioned screws potentially expose the patient to neurologic or visceral injury [12, 13]. Therefore, the ideal use of pedicle screw instrumentation would be a construct that maximizes clinical and radiographic benefit to the patient while minimizing potentially poor outcomes and cost.

The ideal number of pedicle screws that should be used for curve correction in AIS is unclear, and wide variation in clinical practice exists [14, 15]. The concept of anchor density is a useful term for study of the relative number of implants in each spinal fusion construct. Anchor density is defined as number of implants (typically screws or hooks) per vertebral level fused [15]. This can range from 0 (an uninstrumented level) to 2, which would be bilateral implants at a single level. The effect of higher or lower anchor density on the clinical and radiographic outcomes of AIS is unclear. Some studies have shown modest radiographic or patient-reported outcome advantages in high-density pedicle screw constructs [6, 16], while other investigations have shown no radiographic or clinical advantage [17, 18]. However, higher density constructs have been implicated in achieving less postoperative kyphosis, potentially placing the spine at a higher risk of sagittal imbalance. Higher density constructs have also been associated with longer surgical times, more blood loss, and higher cost without demonstrating an improvement in patient satisfaction [17, 18]. Some small studies of low-density constructs have shown favorable outcomes [19]. One investigation showed stable correction at 10-year follow-up with only 50% of all potential anchor sites utilized [20].

The Lenke classification has become the most widely used in the classification of AIS [21]. Some studies have shown no difference in clinical or radiographic outcomes between high- and low-density constructs in Lenke 1 and Lenke 5 AIS [22, 23]. Others have shown some differences in radiographic outcomes but were unable to demonstrate whether these radiographic differences were clinically relevant. Fewer studies have examined the effect of implant density on radiographic outcomes in Lenke 2 AIS. Lenke 2 AIS is defined as both a structural upper thoracic minor curve and a structural main thoracic major curve [21]. To our knowledge, only a single study (Larson et al.) has been published including the effect of anchor density on radiographic outcomes in Lenke 2 AIS [14]. This study demonstrated improved major and minor curve magnitude correction at 2 years postoperatively in high-density constructs. However, the authors were uncertain if this small improvement was clinically significant. Previous literature has demonstrated wide variation in surgeon preoperative correction objectives, as well as in technical selection of correction maneuvers and osteotomies performed [24]. Therefore, we felt additional study may help better establish whether an association exists between anchor density and curve correction in Lenke 2 AIS.

There is potential for this study to add to the field of translational orthopedics. Additional information regarding whether or not higher density screw constructs result in better deformity correction could aid surgeons in selecting the appropriate implant density for posterior spinal fusion in Lenke 2 AIS. Further, selecting a lower density construct may help lower rates of malpositioned implants and associated morbidity. This study suggests that lower implant density may result in similar radiographic outcomes in Lenke 2 AIS.

The purpose of this study was to determine whether higher screw density constructs lead to improved major and minor curve magnitude correction in Lenke 2 AIS. Secondary goals of this study were to identify factors that may predict postoperative curve magnitude and percent curve correction.

## Materials and methods

This was a retrospective study of AIS patients who underwent posterior spinal fusion at a single institution. Patients aged 11–17 years who underwent posterior spinal fusion for AIS between 2007 and 2017 were identified from chart review. All curves were classified according to the Lenke classification for idiopathic scoliosis by a single qualified reviewer. Upper thoracic curves were considered structural if the curve magnitude was greater than 25° using the Cobb measurement technique on preoperative bending films or greater than 40° if preoperative upper thoracic bending films were not obtained based on the high likelihood that a curve of this magnitude would be structural. Patients who underwent fusion of both the upper and main thoracic structural curves (Lenke 2) with at least 2 years of follow-up were included. All patients had either all pedicle screw or pedicle screw and hook constructs. In all cases, the predominant implant was pedicle screw fixation. All patients had intraoperative neuromonitoring, and charts were reviewed for intraoperative and postoperative complications. No postoperative complications were identified in our study group. Exclusion criteria were patients who had prior surgical treatment for scoliosis.

Demographic, surgical, and radiographic data were obtained from the medical record. Demographic data included sex; preoperative weight, height, body mass index, and body mass index percentile for age; past medical history; and duration of follow-up. Surgical data included number of levels fused, number and type of anchors (hooks and screws), implant density (percent anchors placed relative to total available anchor sites in the construct), pedicle screw ratio (percent anchors that are pedicle screws), anchor density (number of anchors/level fused), pedicle coefficient (anchor density multiplied by pedicle screw ratio), and total cost of the anchors used ($500/hook, $800/screw). Radiographic data included pre- and postoperative major structural and minor structural coronal curve magnitudes, preoperative percent curve flexibility [(preoperative curve magnitude−preoperative bending curve magnitude)/preoperative curve magnitude], percent curve correction at first postoperative follow-up and most recent follow-up, correction index (percent correction at most recent follow-up/percent curve flexibility), pre- and postoperative T5-T12 kyphosis, and percent change in T5-T12 kyphosis at first postoperative follow-up and most recent follow-up. Postoperative complications were reviewed for all patients.

Univariable and multivariable regression and elimination analysis were performed. Independent variables included in the regression analysis were sex, age at time of surgery, preoperative curve magnitude, percent curve flexibility, number of levels fused, implant density, pedicle screw ratio, anchor density, and pedicle coefficient. Dependent variables were postoperative major curve magnitude, percent major curve correction, postoperative minor curve magnitude, percent minor curve correction, postoperative thoracic kyphosis, and percent thoracic kyphosis change. After regression analysis, an elimination analysis was performed in which the highest nonsignificant variable (*p*>0.10) in each model was removed, and this process was repeated until only statistically significant variables remained for the outcomes of interest. STATA/MP 14.2 (StataCorp, LLC, College Station, TX) was used for all analyses. The cutoff for significance for all regression analyses was set at *P*=0.10.

## Results

Thirty patients (21 females, 9 males) were analyzed. Demographics are shown in Table 1. Average age at time of surgery was 14.0 ± 1.8 years (range, 11–17 years), and median length of follow-up was 2.8 years (IQR 2.1–4.0 years). Surgical and radiographic data are presented in Tables 2 and 3. At most recent follow-up, mean percent major curve correction was 54 ± 13%, and mean percent minor curve correction was 35 ± 15%. Average implant density was 80 ± 10%, and average anchor density was 1.6 ± 0.2 anchors per level. Pedicle screws consisted of 92 ± 8% of all anchors utilized. Mean total anchor cost was $14,463 ± 2683.

**Table 1:** Patient demographics

Age at time of surgery (years; mean ± SD)	14.0 ± 1.8
Sex (*n* [%] female)	21 (70)
Preoperative weight (kg; mean ± SD)	56.5 ± 15.2
Preoperative height (cm; mean ± SD)	162.6 ± 9.6
Preoperative BMI (kg/m^2^; mean ± SD)	21.3 ± 4.8
Preoperative BMI percentile for age (mean ± SD)	55.0 ± 30.2
Length of follow-up (years; median [IQR])	2.8 (2.1–4.0)

*SD*, standard deviation; *BMI*, body mass index; *IQR*, interquartile range

**Table 2:** Surgical data

Number of levels fused	11.6 ± 1.7
Total number of anchors	18.6 ± 3.0
Implant density (%)	80 ± 10
Pedicle screw ratio (%)	92 ± 8
Anchor density	1.6 ± 0.2
Pedicle coefficient	1.5 ± 0.3
Total anchor cost	$14,463 ± 2683

All values are shown as the mean ± standard deviation

**Table 3:** Radiographic data

	Preoperative		First postoperative follow-up		Most recent postoperative follow-up		
	Curve magnitude (degrees)	Percent flexibility	Curve magnitude (degrees)	Percent change	Curve magnitude (degrees)	Percent change	Correction index
Major curve	63 ± 13	34 ± 10	26±6	58±10	29±9	54±13	2±1
Minor curve	43 ± 8	21 ± 12	27±10	38±17	28±10	35±15	3±3
Thoracic kyphosis	25± 13	n/a	23±13	17±84	22±9	4±87	n/a

All values are shown as the mean ± standard deviation

After controlling for sex, age at time of surgery, preoperative curve magnitude, percent curve flexibility, and number of levels fused, no association was found between implant density, pedicle screw ratio, anchor density, and pedicle coefficient and postoperative major curve magnitude, percent major curve correction, postoperative minor curve magnitude, percent minor curve correction, postoperative thoracic kyphosis, and percent thoracic kyphosis change at most recent follow-up. Although the overall model for major curve magnitude was significant (*P*=0.014), none of the independent variables were significant in the analysis. Regression analysis revealed that female sex was statistically significantly associated with postoperative major curve magnitude [*P*=0.004, *β* coefficient 11.08, 95% CI (4.07–18.09)], percent major curve correction [*P*=0.003, *β* coefficient −18.06, 95% CI (−29.42–6.70)], and thoracic kyphosis [*P*=0.020, *β* coefficient 10.17, 95% CI (1.78–18.56)]. Preoperative major curve magnitude was a significant predictor of postoperative major curve magnitude [*P*=0.010, *β* coefficient 0.40, 95% CI (0.11–0.70)].

Elimination analysis (Table 4) demonstrated that the most important predictors of postoperative major curve magnitude at most recent follow-up were female sex [*P*=0.001, *β* coefficient 9.01, 95% CI (3.80–14.21)] and preoperative major curve magnitude (*P*=0.0001, *β* coefficient 0.39, 95% CI (0.19–0.58)]. Predictors of percent major curve correction at most recent follow-up were female sex [*P*=0.001, *β* coefficient −14.04, 95% CI (−22.21 to −5.86)] and age at time of surgery [*P*=0.084, *β* coefficient −2.00, 95% CI (−4.29–0.29)]. Predictors of postoperative thoracic kyphosis at most recent follow-up were female sex [*P*=0.005, *β* coefficient 9.05, 95% CI (2.99–15.11)] and percentage major curve flexibility [*P*=0.036, *β* coefficient −31.52, 95% CI (−60.77 to −2.27)].

**Table 4:** Results of elimination analysis

	Female sex	Preoperative major curve magnitude	Age at time of surgery	Preoperative percent major curve flexibility
Postoperative major curve magnitude	***P*=0.001** *β* = 9.00, 95% CI (3.80–14.20)	***P*=0.0001** *β* = 0.39, 95% CI (0.19–0.58)		
Percent major curve correction	***P*=0.001** *β* =−14.04, 95% CI (−22.21 to −5.86)		***P*=0.084** *β* = −2.00, 95% CI (−4.29–0.29)	
Postoperative thoracic kyphosis	***P*=0.005** *β* =9.05, 95% CI (2.99–15.11)			***P*=0.036** *β* = −31.52, 95% CI (−60.77 to −2.27)

## Discussion

Our study showed that neither anchor density nor implant density was predictive of postoperative major curve magnitude, minor curve magnitude, or thoracic kyphosis in Lenke 2 AIS. Anchor and implant density were also not predictive of percent major and minor curve correction or change in thoracic kyphosis. Female sex was the strongest predictor of postoperative major curve magnitude and percent major curve correction. In addition, preoperative major curve magnitude was predictive of postoperative major curve magnitude, and age at time of surgery was predictive of percent major curve correction.

The findings of this study contrast somewhat with previously reported outcomes of anchor density in Lenke 2 AIS. To our knowledge, the largest such study reviewing the relationship between anchor density and Lenke 2 curves was published by Larson et al. in 2014 [14]. In that study, high- versus low-density constructs were arbitrarily determined with high anchor density defined as >1.54 screws per level fused. High anchor density was associated with a small but statistically significantly lower postoperative major curve magnitude (25 vs 21° at 2-year follow-up) and higher percent major curve correction (58% vs 65% at 2-year follow-up). Cost data was not reported. The authors did note that it is unclear whether the statistically significant increase in percent major curve correction meets the minimal clinically important difference. The common acceptance of a 5° error in the Cobb measurement technique further calls the significance of this finding into question. High anchor density was also associated with a small but statistically significantly lower T2 to T12 kyphosis at 1-year follow-up (30 vs 20°) but not at 2-year follow-up (31 vs 28°). We chose to evaluate anchor density as a continuous variable. The mean anchor density (1.6 ± 0.2) in our study is similar to Larson et al.’s study [14], but we found no association between anchor density and percent major curve correction or thoracic kyphosis at minimum 2-year follow-up. In contrast to some studies, higher anchor density did not negatively influence postoperative thoracic kyphosis. Our study included fewer surgeons, which contributes to more consistency in operative technique and correction maneuvers compared to a large multicenter study. Previous literature has demonstrated wide variation in surgeon preoperative correction objectives, as well as in technical selection of correction maneuvers.

There are also several limitations to our study. First, not all our patients had preoperative upper thoracic bending films, so we could not assess curve flexibility in those patients. In addition, this study only investigated radiographic outcomes since patient-reported outcomes were not routinely collected during the study period. Previous analysis of Lenke 2 curves demonstrated improved Scoliosis Research Society (SRS) Appearance scores at 1- and 2-year follow-up with higher density constructs. However, no association was noted between higher density constructs and SRS Activity and Satisfaction scores or Spinal Appearance Questionnaire scores [14]. Lastly, we had a small sample of patients, so our study may have been underpowered to detect any association between implant density and radiographic outcomes. This may be in part due to Lenke 2 curves making up approximately 19% of operative AIS curves in one study [25]. However, while Larson et al.’s multicenter study [14] reported statistically significant differences in postoperative major curve magnitude and percent major curve correction, these small differences are not likely clinically significant. More work should be done to determine what magnitude of change might be clinically significant.

## Conclusions

In conclusion, this retrospective study demonstrates that neither anchor nor implant density were associated with major or minor curve magnitude or thoracic kyphosis at 2-year follow-up after posterior spinal fusion for Lenke 2 AIS. Factors that appear to predict postoperative major curve magnitude and percent major curve correction are female sex, preoperative major curve magnitude, and age at time of surgery. In an era of cost-conscious medical care, the ideal implant density for achieving and maintaining curve correction while minimizing cost and exposure of the patient to the potential risks of screw malposition remain to be clarified. Moreover, the minimal clinically important difference for change in radiographic parameters is currently unknown. Our study supports the use of lower implant density constructs in the surgical treatment of Lenke 2 AIS. Further studies are needed to ascertain the ideal implant density to achieve maximal radiographic- and patient-reported outcomes.

## Data Availability

The datasets used and/or analyzed during the current study are available from the corresponding author on reasonable request.

## Abbreviations

PSF: Posterior spinal fusion
AIS: Adolescent idiopathic scoliosis
